# Myocarditis or Pericarditis Following mRNA COVID-19 Vaccination

**DOI:** 10.1001/jamanetworkopen.2022.18512

**Published:** 2022-06-01

**Authors:** Eric S. Weintraub, Matthew E. Oster, Nicola P. Klein

**Affiliations:** Immunization Safety Office, US Centers for Disease Control and Prevention, Atlanta, Georgia; Immunization Safety Office, US Centers for Disease Control and Prevention, Atlanta, Georgia; Children’s Healthcare of Atlanta, Emory University, Atlanta, Georgia; Kaiser Permanente Vaccine Study Center, Kaiser Permanente Northern California, Oakland

Buchan and colleagues^1^ describe findings from a population-based cohort study in Ontario, Canada, that used data from a passive vaccine-safety surveillance system to evaluate reported rates of myocarditis or pericarditis following receipt of an mRNA COVID-19 vaccine, mRNA-1273 (Moderna Spikevax) or BNT162b2 (Pfizer-BioNTech Comirnaty). The authors found that reported rates of myocarditis or pericarditis were higher after vaccination with mRNA-1273 compared with BNT162b2 and that for both vaccines, the rate was higher following dose 2 of the primary 2-dose series when the interdose interval (the timing between dose 1 and dose 2) was 30 days or less. These findings add to the body of knowledge about the association of mRNA COVID-19 vaccination with myocarditis and pericarditis and offer additional insight into the differential risk between the 2 mRNA COVID-19 vaccine products and the possible association of the interdose interval with risk of myocarditis or pericarditis.

Global vaccine-safety monitoring of adverse events following COVID-19 vaccination has been ongoing since COVID-19 vaccines became available in December 2020.^2-4^ Public health and regulatory bodies have been using passive surveillance systems in combination with data on doses administered, clinical reports, and population-based electronic medical record systems to evaluate the association of COVID-19 vaccination with myocarditis and pericarditis.^2-4^ The evidence gathered to date supports an association between mRNA COVID-19 vaccination and myocarditis or pericarditis; the risk appears highest for adolescent and young adult male individuals following dose 2, with symptom onset usually occurring within several days of vaccination.^2-7^

Buchan and colleagues^1^ used a COVID-19 vaccine registry that captures doses administered and an electronic reporting system that collects adverse events following immunizations to further investigate this association. Reported rates of myocarditis or pericarditis were generated by vaccine product, age, sex, and dose number as well as by the interdose interval. In summary, the reported rates for myocarditis and pericarditis among male individuals were higher than those among female individuals for all but 1 age group (25 to 39 years). This finding was consistent for both dose 1 and dose 2 of BNT162b2 and mRNA-1273. Reported rates of myocarditis or pericarditis were higher following dose 2 for both vaccines. The highest reported rate was observed for male individuals aged 18 to 24 years following dose 2 of mRNA-1273; the rate in this age group was more than 6 times higher than the rate following dose 2 of BNT162b2. These primary findings are generally consistent with findings from other surveillance systems across multiple countries.^3,4^

An important new contribution from the study by Buchan and colleagues^1^ is the finding that a longer time interval between dose 1 and dose 2 in the primary mRNA COVID-19 vaccination series may be associated with a lower risk of myocarditis or pericarditis. The overall reported rates of myocarditis or pericarditis following dose 2 were higher for both vaccine products when the interdose interval was 30 days or less compared with 31 to 55 days and 56 days or more. Although the absolute numbers were small, there was a consistent reduction in the rates of myocarditis or pericarditis with increasing intervals between doses, with the lowest rates occurring among individuals with interdose intervals of 56 days or more. In addition, data from other countries indicate that vaccine effectiveness may be higher with an interdose interval for mRNA vaccinations of 6 to 8 weeks compared with the 3- to 4-week interval that is recommended in the US.^5^ Therefore, an 8-week interval may be optimal for some people aged 12 years or older, especially for male individuals aged 12 to 39 years.^5^

Another new contribution of the study by Buchan and colleagues^1^ is the finding that a heterologous second dose with mRNA-1273 (ie, BNT162b2 for dose 1 followed by mRNA-1273 for dose 2) was associated with higher reported rates of myocarditis or pericarditis than was a homologous second does with mRNA-1273 (ie, mRNA-1273 for both dose 1 and dose 2). The reasons for and significance of this finding are unclear, but it merits further study and replication in other data systems. This finding was not replicated when comparing a heterologous vs homologous vaccine series for BNT162b2; the homologous series had slightly higher reported rates of myocarditis or pericarditis for dose 2.

Studies of myocarditis or pericarditis following mRNA COVID-19 vaccination have tended to focus on younger age groups because of their higher risk as observed in several vaccine-safety surveillance systems. Based on an analysis from Vaccine Safety Datalink, an electronic medical record-based monitoring system in the US, mRNA vaccination was associated with a substantially increased risk of myocarditis or pericarditis in persons aged 18 to 39 years, with the highest risk occurring in the 0 to 7 days following dose 2 of mRNA-1273 or BNT162b2.^4^ Additional analysis of Vaccine Safety Datalink data indicated that the risk of myocarditis or pericarditis was higher for mRNA-1273 compared with BNT162b2.^6^ A study conducted in Denmark also found a higher risk for myocarditis or pericarditis following mRNA-1273 vaccination compared with BNT162b2 when evaluating the risk after dose 2 in male individuals aged 12 to 39 years.^7^ The corresponding rates of myocarditis or pericarditis within 28 days of vaccination in the Denmark study was 1.8 per 100 000 vaccinated individuals (95% CI, 0.8-3.4 per 100 000 vaccinated individuals) for BNT162b2 and 9.4 per 100 000 vaccinated individuals (95% CI, 5.0-16.0 per 100 000 vaccinated individuals) for mRNA-1273; dose 1 findings were not reported.

COVID-19 vaccination has prevented substantial morbidity and mortality and has been the most effective primary prevention strategy against COVID-19 infection and serious complications. As the epidemiology of the COVID-19 pandemic continues to evolve and as vaccination programs expand to include younger age groups and additional booster doses, vigilance in monitoring for myocarditis or pericarditis and other adverse events will be critical to ensuring that public health and regulatory authorities have timely and accurate safety data to weigh the benefits and risks of vaccination and make evidence-based recommendations to protect the public and mitigate the pandemic.
